# Supplementary material for: A set of multi-entry identification keys to African frugivorous flies (Diptera, Tephritidae)
Source: Zookeys. 2014 Jul 24;(428):97–108. doi: 10.3897/zookeys.428.7366 (PMC4143993; doi:10.3897/zookeys.428.7366)
Supplement: Supplementary material 6 — Key to Ceratitis [file zookeys-428-097-s006.zip › SF6_ZooKeys_key to Ceratitis/key/SF6_ZooKeys_key to Ceratitis/Media/Html/Ceratitis nana .htm]

Ceratitis (Ceratalaspis) nana Munro


***Ceratitis*** **(*Ceratalaspis*)
*nana* Munro**

*Ceratitis* (*Pardalaspis*) *nana*
Munro, 1933b: 9.

Body
length: 3.20 mm; wing length: 3.50 mm.

Male

Head.
Antenna yellow. Third antennal segment three times as long as second segment.
Arista with short to medium long hairs over entire length. Frons with short
scattered hairs which are largely the same colour as frons; flat, in lateral
view not distinctly projecting forwards at antennal implant. Chaetotaxy normal
for subgenus.

Thorax.
Postpronotum white, spotted. Ground colour of mesonotum pale, with very faint
orange tinge. Chaetotaxy normal for subgenus. Scapular setae pale. One
anepisternal bristle. Scutellum white basally, otherwise yellow with three
black separate markings apically, median spot more or less diamond shaped; two
dark separate spots subbassaly, hardly touching base of scutellum. Subscutellum
with three dark spots, same position as scutellar spots.

Legs
yellow; setation typical for subgenus, mainly pale especially on femora.
Posterior and posterodorsal and ventral rows on fore femur pale.

Wing
bands with markings extensively yellow; cubital band free, medial band absent;
crossvein r-m at middle of discal cell. Crossvein dm-cu posteriorly more
inwards than anteriorly.

Abdomen.
Yellow in ground colour. Weak spotted pattern, no distinct banding.

Female

Unknown.�

(Description
after De Meyer, 1998)
